# Telehealth usefulness during postpartum hemorrhage management: a systematic review

**DOI:** 10.61622/rbgo/2026rbgo71

**Published:** 2026-09-23

**Authors:** Albaro José Nieto-Calvache, Juliana Maya, Francesco D’Antonio, Nerissa Denswil, José Miguel Palacios-Jaraquemada, Eva Pajkrt, María Alejandra Suárez-Revelo, Marcus Rijken

**Affiliations:** 1 Department of Obstetrics and Gynecology Amsterdam Netherlands Department of Obstetrics and Gynecology, Amsterdam, The Netherlands.; 2 Unidad de Alta Complejidad Obstétrica Fundación Valle de Lili Cali Colombia Unidad de Alta Complejidad Obstétrica, Fundación Valle de Lili, Cali, Colombia.; 3 Universidad del Valle Departamento de Ginecologia y Obstetricia Cali Colombia Departamento de Ginecologia y Obstetricia, Universidad del Valle, Cali, Colombia.; 4 Universidad ICESI Department of Health Sciences Cali Colombia Department of Health Sciences, Universidad ICESI, Cali, Colombia.; 5 University of Chieti Center for Fetal Care and High-risk Pregnancy Via dei Vestini Chieti Italy Center for Fetal Care and High-risk Pregnancy, University of Chieti, Via dei Vestini, Chieti, Italy.; 6 Amsterdam University Medical Centers Amsterdam Netherlands Amsterdam University Medical Centers, Amsterdam, The Netherlands.; 7 Hospital Universitario de CEMIC Buenos Aires Argentina Hospital Universitario de CEMIC, Buenos Aires, Argentina.; 8 Centro de Investigaciones Clínicas Fundación Valle de Lili Cali Colombia Centro de Investigaciones Clínicas, Fundación Valle de Lili, Cali, Colombia.; 9 Dutch Cancer Institute Amsterdam Netherlands Dutch Cancer Institute, Amsterdam, The Netherlands.; 10 UMC Utrecht Global Health Amsterdam Netherlands UMC Utrecht Global Health, Amsterdam, The Netherlands.

**Keywords:** Telemedicine, Postpartum hemorrhage, Placenta accreta, High-risk pregnancy, Maternal mortality

## Abstract

**Objective:**

This literature systematic review aims to evaluate tele-medicine's effect in managing postpartum hemorrhage

**Data sources:**

MEDLINE (Ovid), Embase (Ovid), and the Cochrane Library were consulted from inception to March 27, 2024.

**Study selection:**

Studies evaluating tele-medicine interventions (including tele-education, tele-consultation, virtual interventions for improving hospital processes) in the management of postpartum hemorrhage were included, without restrictions on study design.

**Data collection and analysis:**

Data extraction and quality assessment were performed. Due to substantial heterogeneity across studies, a meta-analysis was not conducted, and results were synthesized narratively. Risk of bias was assessed using the Newcastle-Ottawa Scale for cohort studies.

**Results:**

Nineteen studies were included. Most were conducted in low and middle-income countries (n=17/19) and used observational or quasi-experimental design (n=15/19). Four cluster randomized controlled trials evaluated the effectiveness of virtual training strategies, although only in one of them clinical variables were incorporated. Most studies reported improvements in process-related or clinical outcomes following telemedicine interventions. Reported barriers included limited funding, challenges in implementing recommendations at local hospitals, and lack of incentives for sustained use.

**Limitations:**

The included studies were heterogeneous in design, interventions, and outcomes, and most had observational designs, limiting the strength of the evidence.

**Conclusion:**

Developing and maintaining resilient health systems to address postpartum hemorrhage in low-resource settings requires innovative approaches that adapt technology to the context to enhance health outcomes. One such innovation could be tele-medicine, but further research is needed on barriers to implementation and translating the potential benefits of education and timely interaction between patients and expert groups into improved clinical outcomes.

**PROSPERO:** #CRD42023448275

## Introduction

Postpartum hemorrhage (PPH) is the leading cause of maternal death globally, with 90% of cases occurring in low- and middle-income countries (LMIC).^(1,2)^

Maternal mortality in LMIC is driven by multiple delays: limited access to quality care,^(3)^ poor recognition of warning signs by women and families, geographical or financial barriers to primary care, and insufficient referral systems and health personnel.^(4)^

Though multi-level interventions are needed to improve PPH care, tele-medicine offers a simple, low-cost solution by enabling communication between expert groups in regional reference hospitals and primary providers or patients.^(5)^ A specific example of a condition predisposing to a high PPH risk is the Placenta Accreta Spectrum (PAS). While this can present in a spectrum of severity, the key in management to prevent severe PPH or even maternal death lies in early detection during antenatal care or fast recognition and treatment when suspected during surgery. Tele-medicine in PAS care can reduce the incidence of PPH.^(6,7)^

Tele-medicine has improved maternal-fetal care in remote areas.^(3)^ There is robust evidence of its usefulness in managing non-emergent conditions.^(8)^ however, its validity during obstetric emergency care faces some resistance among health professionals who question its safety and effectiveness.^(9)^

In recent years, especially after the COVID-19 pandemic, health professionals have increasingly been using their smartphones to facilitate the care of their patients (m-Health).^(10)^ The use of low-cost or free computer platforms for healthcare activities is becoming popular, even in communities with limited resources.^(11)^

This systematic review aims to evaluate the effect of telemedicine in the management of postpartum hemorrhage.

## Methods

Protocol and registration : This systematic review was registered with PROSPERO (2023 CRD42023448275) and conducted according to the PRISMA statement.^(12)^ No amendments to the protocol were made after registration in PROSPERO.

Eligibility criteria, information sources, search strategy: Any types of studies/designs were eligible for review if they evaluated telemedicine interventions in women with postpartum hemorrhage or at risk of postpartum hemorrhage, and reported clinical or process-related outcomes. Only studies published in English were included. Conference abstracts, studies focusing on children, and animal studies were excluded. The three complete search strategies are available in the supplementary material (Chart 1S-3S). A medical information specialist developed a systematic literature search strategy for MEDLINE (Ovid), Embase (Ovid) and the Cochrane Library (Cochrane Database of Systematic Reviews and Cochrane Trials), focusing on the following two search concepts: 1) acute diagnosis and treatment of PPH and 2) tele-medicine. The search strategy combined controlled vocabulary (MeSH terms) and free-text terms. Search filters were applied to exclude studies focusing on children and animals.

### Study selection

References were managed with EndNote, and deduplicated with DedupEndNote (version 1.0.4) (https://github.com/globbestael/DedupEndNote). Studies were assessed with Rayyan for eligibility by two independent reviewers (A.J. Nieto-Calvache and J. Maya) and disagreements were discussed with a third and fourth reviewer (M. Rijken and E. Pajkrt). The final search was performed on 27 March 2024, with no restrictions on date. Additionally, a manual reference search (snowballing) of the included papers was conducted to identify relevant publications not initially captured. We did not search for trial registries or conference proceedings in this study. As many mHealth interventions may not be published in the peer-reviewed literature, a grey literature search complemented the systematic literature research.

### Data extraction

The information extracted included study design, location, target population/size, supply or not of hardware, intervention or tele-medicine type (tele-education, App use, tele-consultation, tele-support through text messaging, others), activities during the study, results per classification (studies using tele-education and studies using tele-consultation), difficulties (barriers) during tele-medicine strategy implementation.

### Assessment of risk of bias

Quality assessment of the included studies was performed according to the Newcastle-Ottawa Scale (NOS) for cohort studies. NOS allows a maximum of four stars in the selection category, two stars in the comparability category, and three stars in the outcome category, for a total maximum of nine stars.

### Data synthesis

After data collection, the data was evaluated, and considering the excessive heterogeneity of the selected studies, a meta-analysis was not possible, so we presented a systematic review on tele-medicine interventions for the management of PPH. Studies were grouped according to the type of telemedicine intervention (tele-education and tele-consultation) for synthesis. Extracted data were summarized in charts and descriptive formats. This systematic review did not receive any financial funding.

### Statistical analysis

No formal statistical analysis was performed due to heterogeneity among included studies. Therefore, results are presented descriptively using counts and proportions. No sensitivity analyses were performed. Reporting bias was not formally assessed due to the narrative nature of the synthesis and heterogeneity of included studies. The certainty of evidence was not formally assessed.

## Results

### Study selection

The initial search yielded 5,600 records, of those 1,537 were excluded as they were duplicates, leaving 4,063 citations for review. 4,034 references were excluded for not meeting the predefined eligibility criteria. The main reasons for exclusion at this stage included lack of relevance to telemedicine interventions, absence of postpartum hemorrhage outcomes, non-interventional publications (e.g., reviews, editorials), conference abstracts without evaluable data, studies conducted in non-obstetric populations, and duplicate records not previously identified. 29 full-text articles were assessed figure 1, of which 19 included in the final review chart 1.^(13-31)^ Not additional results were obtained from the grey literature review.

**Figure 1:**
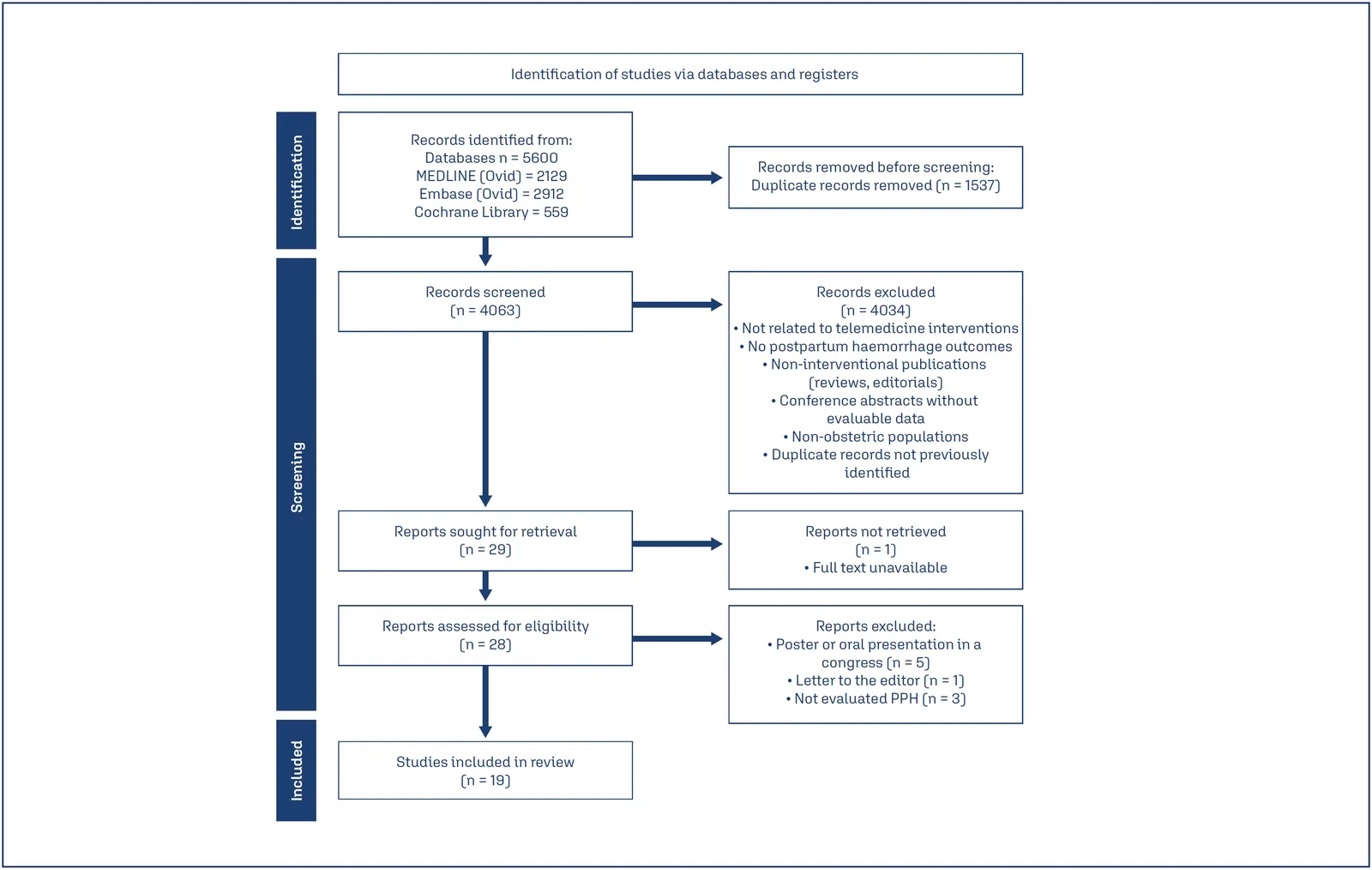
PRISMA 2020 flow diagram outlining study selection process

Source: Adapted from Page MJ, McKenzie JE, Bossuyt PM, et al. The PRISMA 2020 statement: an updated guideline for reporting systematic reviews. BMJ. 2021;372:n71. doi:10.1136/bmj.n71 ^(12)^

**Chart 1:** General characteristics of the included studies

Author/Year	Country	Study design	Hardware supply	Intervention	Activities during study
Aitken et al. 2014^(13)^	Canada	Case Report	No	Tele-consultation	Referral after intraoperative identification of PAS^‡^
Rahman et al. 2017^(14)^	Bangladesh	Pre-Post Intervention Design	Not Applicable	Organization of the transfusion service. App Use	Assessment of the effectiveness of an online blood transfusion information management application
Bolan et al 2018^(15)^	Congo	Cluster RCT pilot study	Yes, cellphones	Education. App Use	Virtual training in obstetric emergency management activities using a medical app (SDA)
Mbaruku et al. 2018^(16)^	Tanzania	Cohort Prospective	Yes, cellphones	Tele-consultation. Text messaging	In person education followed by use of Closed User Group mobile phone network within the overall EmOC project.
Sun et al. 2018^(17)^	China	Cohort Retrospective	No	Tele-consultation. Text messaging	Online-to-offline model for PAS management
Amoakoh et al. 2019^(18)^	Ghana	Cluster RCT	Yes, cellphones	Tele-consultation. Text messaging	Access to the Medical advice service during emergencies. It involved a clinical decision-making support system that utilized text messaging.
Klokkenga et al. 2019^(19)^	Ghana	Cluster RCT	Yes, cellphones	Education. App use	Virtual training in obstetric emergency management activities using a medical app (SDA)
Nieto-Calvache et al. 2021^(20)^	Colombia	Cohort Prospective	No	Tele-consultation	"Tele-mentory"
Nieto-Calvache et al. 2021^(21)^	Colombia	Cohort Retrospective	No	Education and Tele-consultation	Virtual education and tele-guidance during PAS^‡^ surgery
Nishimwe et al. 2021^(22)^	Rwanda	Pre-Post Intervention Design	Yes, cellphones	Education. App Use	Virtual training in obstetric emergency management activities using a medical app (SDA)
Escobar et al. 2022^(23)^	Colombia	Pre-Post Intervention Design	Yes, tablet, screen	Tele-consultation	In-person education followed by long term virtual support
Nieto-Calvache et al. 2022^(24)^	Colombia	Cohort Prospective	No	Tele-consultation	Tele-consultation for PAS^‡^ management
Nieto-Calvache et al. 2022^(25)^	Colombia	Cohort Prospective	No	Education	Virtual education and tele-guidance during surgery for implementation of a new surgical technique
Nieto-Calvache et al. 2022^(26)^	Colombia	Case series	No	Tele-consultation	Tele-consultation during unsuspected PAS^‡^ diagnosis at medium complexity hospitals
Nishimwe et al. 2022^(27)^	Rwanda	Pre-Post Intervention Design	Yes, cellphones	EducationApp use	Virtual training in obstetric emergency management activities using a medical app (SDA)
Aryananda et al. 2023^(28)^	Indonesia, Colombia	Case Series	No	Tele-consultation	Urgent tele-consultation during intraoperative identification of PAS^‡^
Christiansen.2023^(29)^	Ethiopia	Cluster RCT	Yes, cellphones	Education. App use	Virtual training in obstetric emergency managment activities using a medical app (SDA)
Escobar. 2024^(30)^	Colombia	Pre-Post Intervention Design	No	Tele-consultation	In person education followed by long term virtual support and periodics virtual academic activities
Theiler. 2024^(31)^	USA	Case Series	Yes, two-way synchronous audio/visual robotic devices. Point of care ultrasound machines	Tele-consultation	Real time tele-consultation during obstetrics emergencies

### Study characteristics

Six were retrospective studies (four case reports or series, two cohort studies), and 13 were prospective (four cohort studies, five pre-post intervention design studies, and four cluster randomized controlled trials) chart 1. Twelve studies evaluated the impact of tele-consultation on clinical outcomes or quality indicators, such as frequency of virtual support usage, application of a new surgical technique, and uterine preservation PAS. Seven studies evaluated tele-education in PPH Chart 2. Six studies used apps for PPH management. Five used the Safe Delivery App^(29)^ for Virtual training in obstetric emergencies^(15,19,22,27,29)^, evaluating the change in knowledge of trained staff^(15,22)^ and the incidence of PPH before and after the educational process^(19)^ or compared to hospitals where training facilitated by the App was not used.^(27,29)^ The sixth study evaluated the effect of an online blood information management application on the speed of dispatching blood components once transfusion was indicated.^(14)^ One study describe virtual training for the learning of uterine sparing surgery for the treatment of PAS.^(25)^

**Chart 2:** Studies using tele-education in PPH

Author / Year	Participants	Tele-medicine form	Duration	Education activities	Other activities	Follow-up	Results	Identified barriers
Bolan et al. 2018^(15)^	8 hospitals, 62 healthcare workers (46 clinical nurses and midwives, 16 medical doctors), 32 in intervention and 30 in control Rural hospitals	App use	3 months, no exact data on follow-up time	Half-day training session on the use of the SDA Nurses and midwives were expected to use the SDA as often as they wished.	SDA was pre-downloaded to Android smartphones in the DRC capital, Kinshasa, due to poor Internet connectivity in the pilot region was allocated per facility Solar panel battery chargers were given to all 8 facilities with the smartphones to ensure consistent ability to charge.	Eighteen qualitative interviews were conducted with app users and key stakeholders to assess feasibility and acceptability of mLearning and the use of the SDA. Visiting intervention and control facilities weekly to remind providers to use the app	Increase in PPH knowledge scores Variation pre-post intervention of 18.9 in the intervention group vs 1.6 in the control group (out of 100)	Poor practice environment, lack of consistent medications, equipment, and electricity to apply the recommendations from the App Absence of incentives to incorporate an additional activity (consulting the App) to previous ones. Poor economic remuneration
Klokkenga et al. 2019^(19)^	15 hospitals, 146 nurses. Intervention: 70 midwives, seven hospitals, 1,665 deliveries. Controls: 76 midwives, eight hospitals, 1,746 deliveries Rural hospitals	App use	Not clear	After randomization, the midwives in the intervention arm were given a smartphone with the SDA and they watched the videos once while the principal investigator was present. Thus, all midwives watched the app at least once.	No	End of Study Questionnaire filled by the midwives at the trial end, 83% of midwives in the intervention group responded they had watched the videos more than five times	No differences in PPH incidence, severe PPH and volume of blood loss between groups. The validity of the blood loss data was a concern since the data collection was not completely uniformly and this imprecision could not be evaluated	Not reported
Nieto-Calvache et al. 2021^(20)^	1 hospital, 89 PAS patients (76 patients after tele-medicine intervention) Urban hospital	Phone call and text messages direct two-way communication	5 years	Virtual education between an expert center and another hospital in training.	Tele-radiology for diagnosis and surgical planning, and tele-guidance during PAS surgery Medium term tele-mentoring for debriefing cases attended and quality improvement	Continuous contact through easy-access virtual channels (phone calls, instant messaging chat, video calls through free applications) with the expert group who supervised surgical performances and supported improvement initiatives at the local hospital	Better clinical results (More patients treated with uterine-sparing surgery and neuraxial anesthesia, individualized use of REBOA, and cell saving techniques.) in PAS patients over time	Not reported
Nishimwe et al. 2021^(22)^	2 hospitals, 54 participants (33 midwives and 21 nurses) Rural hospitals	App use	6 months	Half-day training session on the use of the SDA Nurses and midwives were expected to use the SDA as often as they wished.	Each of the study hospitals was allocated three smartphones, with pre-installed SDA	Self-administered questionnaires at baseline and at 6 months post-intervention	Difference in PPH knowledge of 17.1% out of 100, with a 2.6% difference in PPH skills.	Gaps in the environmental context and resources
Nieto-Calvache et al. 2022^(25)^	6 hospitals, 6 patients Urban hospitals	Phone call and text messages direct two-way communication	3 months	Virtual training program for the implementation of one step conservative surgery	Baseline observation of the skills or prior knowledge of the participants Theoretical and video based review of the crucial steps of surgery Joint evaluation of a PAS case by reviewing ultrasound images Tele-presence during surgery Debriefing and learning activities	No	The new surgical technique was implemented in all hospitals.	Not reported
Nishimwe et al. 2022^(27)^	2 hospitals, 54 nurses. 114 patients Rural hospitals	App use	14 months 6 months initial evaluation 2 months of intervention with the App 6 months post-intervention	Half-day training session on the use of the App Nurses and midwives were expected to use the SDA as often as they wished.	Each of the study hospitals was allocated three smartphones, with pre-installed App. Participants who owned smartphones, were encouraged to download the App for use during the training.	Two visits per week by research assistants to monitoring the App use by nurses and midwives.	Stable outcomes following PPH management in 78% PPH cases at baseline and 94% at endline. Transferred (unstable) women were 19 and 6% at baseline and endline respectively. Death attributed to PPH appeared in 3% women for the baseline group only	Not reported
Christiansen et. Al. 2023^(29)^	2 hospitals, 13 health centers, 58 health post. 130 midwives and health extension workers	App use	12 months	A half-day introduction to the use of the phone and the SDA was given. 4 cartoon videos about active management of the third stage of labour to prevent PPH, the management of manifest PPH, the removal of a retained placenta and neonatal resuscitation	No	6 and 12 months follow up evaluations using Key Feature Questionnaire prepared with case-based questions related to clinical situations	The diference in increase in scores between the intervention and the control group was signifcant for all the time periods assessed, indicating an efect of the SDA of a more than doubling of the skills scores from baseline	Success probably depends on contextual factors such as strategies for dissemination for the App and support by national health authorities

PPH: Postpartum hemorrhage. SDA: Safe Delivery App. PAS: placenta accreta spectrum. REBOA: Resuscitative Endovascular Balloon Occlusion of the Aorta

Twelve studies reported using tele-consultation during the management of PPH patients or those at risk of it chart 3. In six of these studies, tele-consultation was preceded by in-person^(16,21,23,29)^ or virtual^(18)^ education activities. One Chinese study evaluated an online PAS management model using a WeChat group for direct patient-provider communication, improving hospital stay and hysterectomy rates.^(17)^ Nine studies included the provision of hardware (mobile phones, tablets, or robotic audio/visual communication devices) to participants chart 1.

### Risk of bias of included studies

The results of the quality assessment using the NOS scale tool (with a maximum score of 9) for the included studies are presented in chart 4 (see Supplementary). NOS scores ranged from six to eight in the three cluster RCTs and five to eight in quasi-experimental studies. Overall, the included studies exhibited a medium to high score concerning the selection and comparability of study groups and the ascertainment of the outcome of interest. The major limitation was the heterogeneity in observed outcomes. NOS scores ranged from four to eight in the nine observational studies, with wide variability in the evaluated results.

**Chart 4:** Methodological assessment of included studies based on Newcastle – Ottawa Score (NOS*)

Author / Year	Selection	Comparability	Outcome	Type study
Aitken, 2014^(13)^	Not applicable	Not applicable	Not applicable	Observational
Rahman, 2017^(14)^	★★★	★	★★	Pre-Post Intervention
Bolan, 2018^(15)^	★★★	★★	★★★	Cluster RCT^†^
Mbaruku, 2018^(16)^	★★	★★	★★	Observational
Sun, 2018^(17)^	★★	★	★★	Observational
Amoakoh, 2019^(18)^	★★	★★	★★	Cluster RCT^†^
Klokkenga, 2019^(19)^	★★★	★★	★★	Cluster RCT^†^
Nieto-Calvache, 2021^(20)^	★★★	★★	★★★	Observational
Nieto-Calvache, 2021^(21)^	★★	★	★★	Observational
Nishimwe, 2021^(22)^	★★★	★★	★★	Pre-Post Intervention
Escobar, 2022^(23)^	★★★	★★	★★	Pre-Post Intervention
Nieto-Calvache, 2022^(24)^	★★★	★★	★★★	Observational
Nieto-Calvache, 2022^(25)^	★★★	★★	★★★	Observational
Nieto-Calvache, 2022^(26)^	★	★	★★	Observational
Nishimwe, 2022^(27)^	★★	★	★★	Pre-Post Intervention
Aryananda, 2023^(28)^	★★★	★★	★★★	Observational
Christiansen,2023^(29)^	★★★	★★	★★★	Cluster RCT^†^
Escobar, 2024^(30)^	★★★	★★	★★★	Pre-Post Intervention
Theiler, 2024^(31)^	★★	★★	★★	Observational

### Synthesis of the results

Nineteen studies evaluated the use of tele-medicine in managing PPH chart 1, focusing on two interventions: tele-education to enhance the skills of professionals handling women with PPH or related conditions (specifically PAS), and tele-consultation during the management of PPH cases. The variability in designs and interventions assessed makes a group analysis of the results impossible. Almost all studies showed a benefit in the evaluated outcome after utilizing tele-medicine. One cluster RCT showed no difference in the incidence of PPH between the groups being assessed.^(19)^ Seven studies utilized tele-education chart 2; five of them employed an application designed to teach basic skills to handle emergencies during childbirth, while two focused on the interaction between an expert group executing uterine-sparing surgery in PAS patients and other groups interested in training in that type of procedure. The five studies evaluating the impact of the Safe Delivery App were conducted in rural hospitals in Africa, where the incidence of PPH was not affected.^(19,22,29)^ However, an improvement in knowledge scores among health workers and better clinical outcomes for patients with PPH^(27)^ in hospitals randomized to using the App was observed.^(15)^ Twelve studies used tele-consultation during PPH management (Chart 3). Four studies stemmed from in-person training sessions in low and medium-complexity hospitals,^(16,23,30,31)^ offering medium-term support using tele-consultation during PPH management, resulting in decreased transfusion frequency,^(23)^ adherence to the recommendation of using the non-pneumatic anti-shock garment^(16)^ and reduction of maternal mortality.^(30)^ Two third study originated from in-person training in PAS surgical management, followed by 22 months of interaction between an expert group and a training hospital, using tele-radiology for joint analysis of MRI images and tele-mentoring to plan and execute surgeries, successfully implementing uterine-sparing surgery in a Colombian hospital.^(20,21)^ Three studies describe the utility of urgent tele-consultation during the unexpected diagnosis of PAS,^(13,26,28)^ with favorable outcomes if the surgeon encountering intraoperative PAS decides to seek virtual advice in a tele-consultation and follows the instructions (ideally deferring surgery and referring to a better-resourced hospital). This type of ‘informal’ consultation uses free or low-cost mobile applications and phone calls, which were also described as useful among doctors during non-emergency PAS management in Latin America.^(24)^ One study evaluated the frequency of access to a medical advice service during emergencies in a cluster RCT across 16 rural hospitals^(18)^, finding a decreasing utilization of the service over the 18-month evaluation period, with the virtual advice service used in 44.9% of PPH cases at 6 months, 39.8% at 12 months, and 15.2% at 18 months. Barriers to tele-medicine implementation detailed in charts 2 and 3 include: limited funding for hardware, software, and internet challenges in enabling local hospitals to apply tele-consultation or educational recommendations; and lack of incentives for continued tele-consultations or tele-education use after program launch.

### Reporting bias

Formal reporting bias assessment was not performed due to the narrative nature of the synthesis and the heterogeneity of included studies.

### Certainty of evidence

Formal certainty of evidence assessment was not performed due to substantial heterogeneity in study designs and outcomes. Methodological quality of individual studies was evaluated using the Newcastle-Ottawa Scale chart 4.

## Discussion

### Principal findings

This review found only 19 studies evaluating tele-medicine for PPH management, including emergent cases and scheduled care for high-risk patients, particurarly with PAS. Most (n=17/19) were conducted in low- and middle-income countries with an observational or quasi-experimental design (n=15/19). Four cluster RCTs assessed virtual training (e.g.,using the “safe delivery application” or a medical advice service), but only one incorporated clinical variables.^(18)^

### Comparison with existing literature

PPH remains the leading cause of maternal mortality,^(1)^ highlighting the necessity for every healthcare professional to receive adequate training in its prevention, diagnosis, and treatment. Furthermore, multiple effective therapeutic options are available to halt bleeding and improve outcomes.^(32)^ However, many healthcare workers either lack sufficient training during their professional education^(33)^ or access to continuous medical education to maintain competencies and internalize new recommendations. This problem underscores the importance of standardized management using cognitive aids, teamwork, and institutional support^(34)^ Tele-medicine helps bridge the gap between ideal and actual PPH care.^(35)^ However, our systematic review indicates that the solution is not as straightforward. The challenges faced by healthcare workers vary in each region, and although the COVID-19 pandemic increased acceptance of tele-medicine, incorporating new processes into ingrained practices that have been performed for years is not easy. Our results confirm persisting difficulties for the acceptance of tele-medicine by some healthcare professionals, considerations about the necessary economic investment to initiate the project (hardware, internet connectivity),^(23,31)^ provision of hospitals to execute the recommendations received during tele-consultations,^(16,18)^ and long-term economic sustainability.^(23,31)^

Publications evaluating tele-education for PPH focused on independent study by healthcare professionals^(15,22)^, facilitated by virtual content designed by experts (apps use), or distant mentoring by groups with higher training.^(16)^ Health workers learning via mobile devices is increasing rapidly; however, there is little evidence of effective learning. Five studies assessed the utility of an application (Safe Delivery App) on health workers’ knowledge and practices^(15,19,22,27,29)^ The experience in hospitals in Rwanda showed that nurses in rural hospitals who used the Safe Delivery App improved their knowledge and skills related to PPH, resulting in better clinical outcomes for patients with PPH after the educational intervention. However, cluster RCTs evaluating the use of the Safe Delivery App failed to show differences in clinical outcomes between hospitals randomized to education using this application or standard management,^(19)^ even though there were significant differences in knowledge assessments of the health workers involved with the Safe Delivery App.^(15)^

Demonstrating clinical impact through a single intervention is challenging in a condition affected by numerous variables like PPH. A recent review assessing the effectiveness of mHealth interventions targeting healthcare workers to improve pregnancy outcomes could not demonstrate an effect.^(36)^ The studies included in this systematic review suggest that the best strategy for an educational intervention to translate into better clinical outcomes is medium- to long-term supervision,^(20,30)^ with behavioral interventions^(21)^ such as additional activities reinforcing the newly taught practices, periodic positive reinforcement,^(20)^ frequent feedback of results, and involvement of the various stakeholders in obstetric care in the region.^(30)^ These findings are consistent with in-person interventional studies aimed at reducing morbidity due to postpartum hemorrhage (PPH)^(37,38)^ and reinforce the need to ensure the longitudinal sustainability of telehealth interventions.

The issue of a current knowledge deficit concerning PPH among healthcare workers in LMIC is highlighted in a study from Rwanda,^(22)^ where despite the majority (76%) of participants attending more than ten deliveries per month, the pre-intervention assessment obtained correct responses in fewer than half of the questions. In this review, virtual education was a potential solution to nurses’ and midwives’ challenges in accessing up-to-date learning resources at LMIC. The positive variation of 17 points (out of 100) through independent learning using a mobile application^(22)^ is consistent with another study in Senegal showing sustained improvement in knowledge about family planning using an m-Learning tool.^(39)^

The app-related educational intervention likely requires a more effective implementation strategy. Rather than being a stand-alone tool to improve outcomes, the virtual education strategy might function better as an integrated part of other efforts to enhance the quality of delivery of care. Previous studies clarify that medium- to long-term behavioral interventions facilitate adult learning and behavior change.^(37,38)^ Even the use of beneficial support strategies such as tele-consultation or access to medical advice services rapidly declines over time from the initial strategy of promotion. In a study conducted in Ghana,^(18)^ rural hospitals were provided access to multiple virtual support modalities that were previously unavailable (such as phone calls and short text message services among project members, access to service data for standard emergency obstetric protocols, and access to the internet), but the usage decreased significantly. It started at 44% within six months of the intervention initiation and dropped to 15% at 18 months. Another study in the United States of America also reports very low usage of tele-consultation services for obstetric emergency care, with only 25 tele-consultations over 36 months, despite a high initial economic investment in equipping 17 rural hospitals with robotic communication devices and, in some cases, ultrasound equipment^31^. There are multiple reasons for the declined usage of virtual support over time, one being the so-called “novelty effect”^(40)^ in which new technology is more frequently used when introduced. This improvement in usage isn't necessarily due to actual learning or achievement enhancement, but rather because of increased interest in the new technology, and often, there is a progressive abandonment of adherence to the new procedure. Another reason for the decline in usage of tele-medicine over time could be the discrepancy between protocols and reality on the ground, such as the lack of equipment or resources necessary for effective implementation. This discrepancy between the prescribed protocols or guidelines and the practical limitations faced in real-life situations can deter sustained usage or adoption of virtual support systems.^(41)^ That's why educational interventions and virtual support must be accompanied by administrative and financial support to replenish supplies, streamline processes, and support local leaders responsible for ensuring compliance with recommendations^(15,16,18)^ and adherence to protocols. Similarly, clinical practice guidelines should consider the realities of settings with limited resources and include among their recommendations the application of effective, low-cost, and reproducible strategies in such scenarios.^(42)^

Most maternal deaths due to PPH occur in settings with limited resources, where healthcare investment and the density of doctors and nurses are insufficient. The interaction between hospitals with varying resource availability, involving an initial phase of in-person, virtual, or mixed training, followed by medium to long-term collaboration, has shown benefits in terms of morbidity and mortality. Experiences such as those in California^(43)^ and four countries in Africa^(37)^ underscore that multifactorial interventions with supervision and support are necessary to change local practices. Tele-medicine is likely to be an essential element in such strategies, but this premise needs formal evaluation.

While this systematic Review is the first comprehensive overview of the utility of tele-medicine in managing PPH, it was impossible to conduct a meta-analysis due to the heterogeneity in study designs, populations, and objectives among the identified studies. Furthermore, a significant number of the articles included were previously published by our group. However, most included studies report favorable outcomes with the use of tele-medicine, improving participants’ competencies in educational activities,^(15,22,29)^ facilitating coordination among different actors in healthcare delivery,^(13,17,24)^ or directly impacting clinical outcomes,^(23,30)^ especially in LMICs^(20,28)^ but also in some scenarios with limited resources in high-income countries.^(13,31)^ This suggests a positive effect of tele-health in the management of PPH.

Although the Newcastle–Ottawa Scale allowed a standardized assessment across heterogeneous designs, the absence of design-specific tools such as RoB 2 for randomized trials and ROBINS-I for non-randomized intervention studies may have limited the depth of the risk-of-bias evaluation.

As in any review, some studies may have been missed. Although we limited our search to English, we believe this did not affect the impact of our conclusions. Actually, most of the studies included were conducted in countries with languages other than English (eg Latin America). Additionally, the first article identified was published only ten years ago, with more than half of the studies identified in this Review corresponding to the last four years. It is likely that in the coming years, more publications will appear on experiences with the use of tele-health in PPH, and the results of future systematic reviews or meta-analyses may differ. For now, it is evident that tele-medicine is a promising strategy, but further research is required. Two fundamental elements for tele-health that should be included in future studies are the financial aspects of compensating tele-experts and the legal details of sensitive patient information flow and interaction between physicians across different countries, or even within the same country, in states with different regulations.^(44)^

Tele-medicine is a more cost-effective way to provide healthcare than in-person care, especially when there are geographical, economic, or administrative limitations for patients or medical groups to physically convene or in emergent situations, such as the unexpected intraoperative finding of PAS. Our Review demonstrates a vast majority of positive outcomes in the evaluated variables and calls for the design and implementation of innovative solutions for the persisting issues leading to maternal deaths, especially in low-resource scenarios. The analysis of studies included in this systematic review can impart important lessons about project implementation of new technologies, such as the preference for on-site training in resource-limited settings and for access to tele-medicine on personal mobile phones (m-health), the significance of educating nursing staff as well as physicians, and the addition of medium to long-term follow-up to any educational strategy.

## Conclusion

Resilient PPH care in low-resource settings requires context-adapted innovations like tele-medicine. More research is needed to translate its educational and consultative potential into improved outcomes.

## Data Availability

The research data are described in the article presented.

## References

[1] Nelson JR, Ess RH, Dickerson TT, Gren LH, Benson LS, Manortey SO (2022). Strategies to increase rural maternal utilization of skilled health personnel for childbirth delivery in low- and middle-income countries: a narrative review. Glob Health Action.

[2] Hobbs AJ, Moller AB, Kachikis A, Carvajal-Aguirre L, Say L, Chou D (2019). Scoping review to identify and map the health personnel considered skilled birth attendants in low-and-middle income countries from 2000-2015. PLoS One.

[3] Moyer CA, Johnson C, Kaselitz E, Aborigo R, Ametepi P, Williams J (2017). Using social autopsy to understand maternal, newborn, and child mortality in low-resource settings: a systematic review of the literature. Glob Health Action.

[4] Nieto-Calvache AJ, Palacios-Jaraquemada JM, Vergara-Galliadi LM, Matera L, Sanín-Blair JE, Rivera EP (2021). Latin American Group for the Study of Placenta Accreta Spectrum. All maternal deaths related to placenta accreta spectrum are preventable: a difficult-to-tell reality. AJOG Glob Rep.

[5] Jean-Francois B, Bailey Lash T, Dagher RK, Green Parker MC, Han SB, Lewis Johnson T (2021). The Potential for Health Information Technology Tools to Reduce Racial Disparities in Maternal Morbidity and Mortality. J Womens Health (Larchmt).

[6] Liddy C, McKellips F, Armstrong CD, Afkham A, Fraser-Roberts L, Keely E (2017). Improving access to specialists in remote communities: a cross-sectional study and cost analysis of the use of eConsult in Nunavut. Int J Circumpolar Health.

[7] Cantor AG, Jungbauer RM, Totten AM, Tilden EL, Holmes R, Ahmed A (2022). Telehealth Strategies for the Delivery of Maternal Health Care: A Rapid Review. Ann Intern Med.

[8] Odibo IN, Wendel PJ, Magann EF (2013). Telemedicine in obstetrics. Clin Obstet Gynecol.

[9] Kinoshita S, Kishimoto T (2022). Current Status and Challenges of the Dissemination of Telemedicine in Japan after the start of the COVID-19 pandemic. Telemed J e-Health.

[10] Nieto-Calvache AJ, Maya J, Vergara Galliadi LM, Nieto Calvache AS (2022). Low-cost or free access virtual platforms utility in placenta accreta spectrum. AJOG Glob Rep.

[11] Haleem A, Javaid M, Singh RP, Suman R (2021). Telemedicine for healthcare: Capabilities, features, barriers, and applications. Sens Int.

[12] Page MJ, Moher D, Bossuyt PM, Boutron I, Hoffmann TC, Mulrow CD (2021). PRISMA 2020 explanation and elaboration: updated guidance and exemplars for reporting systematic reviews. BMJ.

[13] Aitken K, Cram J, Raymond E, Okun N, Allen L, Windrim R (2014). “Mobile” medicine: a surprise encounter with placenta percreta. J Obstet Gynaecol Can.

[14] Rahman A, Akhter S, Nisha MK, Islam SS, Ashraf F, Rahman M (2017). Can mHealth improve access to safe blood for transfusion during obstetric emergency?. Int J Womens Health.

[15] Bolan NE, Sthreshley L, Ngoy B, Ledy F, Ntayingi M, Makasy D (2018). mLearning in the Democratic Republic of the Congo: A Mixed-Methods Feasibility and Pilot Cluster Randomized Trial Using the Safe Delivery App. Glob Health Sci Pract.

[16] Mbaruku G, Therrien MS, Tillya R, Mbuyita S, Mtema Z, Kinyonge I (2018). Implementation project of the non-pneumatic anti-shock garment and m-communication to enhance maternal health care in rural Tanzania. Reprod Health.

[17] Sun W, Yu L, Liu S, Chen Y, Chen J, Wen SW (2018). Comparison of maternal and neonatal outcomes for patients with placenta accreta spectrum between online-to-offline management model with standard care model. Eur J Obstet Gynecol Reprod Biol.

[18] Amoakoh HB, Klipstein-Grobusch K, Grobbee DE, Amoakoh-Coleman M, Oduro-Mensah E, Sarpong C (2019). Using Mobile Health to Support Clinical Decision-Making to Improve Maternal and Neonatal Health Outcomes in Ghana: Insights of Frontline Health Worker Information Needs. JMIR Mhealth Uhealth.

[19] Klokkenga CM, Enemark U, Adanu R, Lund S, Sorensen BL, Attermann J (2019). The effect of smartphone training of Ghanaian midwives by the Safe Delivery application on the incidence of postpartum hemorrhage: A cluster randomised controlled trial. Cogent Med.

[20] Nieto-Calvache AJ, Palacios-Jaraquemada JM, Vergara-Galliadi LM, Nieto-Calvache AS, Zambrano MA, Burgos-Luna JM (2021). Training facilitated by interinstitutional collaboration and telemedicine: an alternative for improving results in the placenta accreta spectrum. AJOG Glob Rep.

[21] Nieto-Calvache AJ, Zambrano MA, Herrera NA, Usma A, Bryon AM, Benavides Calvache JP (2021). Resective-reconstructive treatment of abnormally invasive placenta: inter Institutional Collaboration by telemedicine (eHealth). J Matern Fetal Neonatal Med.

[22] Nishimwe A, Ibisomi L, Nyssen M, Conco DN (2021). The effect of an mLearning application on nurses’ and midwives’ knowledge and skills for the management of postpartum hemorrhage and neonatal resuscitation: pre-post intervention study. Hum Resour Health.

[23] Escobar MF, Echavarria MP, Vasquez H, Nasner D, Ramos I, Hincapié MA (2022). Experience of a telehealth and education program with maternal and perinatal outcomes in a low-resource region in Colombia. BMC Pregnancy Childbirth.

[24] Nieto-Calvache AJ, López-Girón MC, Nieto-Calvache AS (2022). The usefulness of inter-institutional collaboration (teleconsultation, eHealth) in the management of placenta accreta. J Matern Fetal Neonatal Med.

[25] Nieto-Calvache AJ, Palacios-Jaraquemada JM, Aguilera LR, Arriaga W, Colonia A, Aryananda RA (2022). Telemedicine facilitates surgical training in placenta accreta spectrum. Int J Gynaecol Obstet.

[26] Nieto-Calvache AJ, Velásquez P, Aguilera R, Aryananda RA, Hidalgo A (2022). Placenta accreta spectrum: intraoperatory analysis for immediate tele-help. J Matern Fetal Neonatal Med.

[27] Nishimwe A, Ibisomi L, Nyssen M, Conco DN (2022). The effect of a decision-support mHealth application on maternal and neonatal outcomes in two district hospitals in Rwanda: pre - post intervention study. BMC Pregnancy Childbirth.

[28] Aryananda RA, Nieto-Calvache AJ, Duvekot JJ, Aditiawarman A, Rijken MJ (2023). Management of unexpected placenta accreta spectrum cases in resource-poor settings. AJOG Glob Rep.

[29] Christiansen AH, Sørensen BL, Boas IM, Bedesa T, Fekede W, Nielsen HS (2023). The impact of the Safe Delivery Application on knowledge and skills managing postpartum haemorrhage in a low resource setting: a cluster randomized controlled trial in West Wollega region, Ethiopia. Reprod Health.

[30] Escobar MF, Echavarría MP, Carvajal JA, Lesmes MC, Porras AM, Mesa V (2024). Hospital padrino: a collaborative strategy model to tackle maternal mortality: a mixed methods study in a middle-income region. Lancet Reg Health Am.

[31] Theiler RN, Torbenson V, Schoen JC, Stegemann H, Heaton HA, Kozhimannil KB (2024). Virtual obstetric hospitalist support for obstetric emergencies and deliveries: the Mayo Clinic experience. Telemed J e-Health.

[32] Hofmeyr GJ (2023). Novel concepts and improvisation for treating postpartum haemorrhage: a narrative review of emerging techniques. Reprod Health.

[33] Nieto-Gutierrez W, Taype-Rondan A (2023). Self-perceived competence in managing obstetric emergencies among recently graduated physicians from Lima, Peru. BMC Med Educ.

[34] Main EK, Goffman D, Scavone BM, Low LK, Bingham D, Fontaine PL, National Partnership for Maternal Safety, Council on Patient Safety in Women's Health Care (2015). National Partnership for Maternal Safety: consensus bundle on obstetric hemorrhage. Obstet Gynecol.

[35] Jayanna K, Mony P, Ramesh BM, Thomas A, Gaikwad A, Blanchard JH (2014). Assessment of facility readiness and provider preparedness for dealing with postpartum haemorrhage and pre-eclampsia/eclampsia in public and private health facilities of northern Karnataka, India: a cross-sectional study. BMC Pregnancy Childbirth.

[36] Amoakoh-Coleman M, Borgstein AB, Sondaal SF, Grobbee DE, Miltenburg AS, Verwijs M (2016). Effectiveness of mHealth interventions targeting health care workers to improve pregnancy outcomes in low- and middle-income countries: a systematic review. J Med Internet Res.

[37] Gallos I, Devall A, Martin J, Middleton L, Beeson L, Galadanci H (2023). Randomized trial of early detection and treatment of postpartum hemorrhage. N Engl J Med.

[38] Althabe F, Buekens P, Bergel E, Belizán JM, Campbell MK, Moss N, Guidelines Trial Group (2008). A behavioral intervention to improve obstetrical care. N Engl J Med.

[39] Diedhiou A, Gilroy KE, Cox CM, Duncan L, Koumtingue D, Pacqué-Margolis S (2015). Successful mLearning Pilot in Senegal: Delivering Family Planning Refresher Training Using Interactive Voice Response and SMS. Glob Health Sci Pract.

[40] Taki S, Lymer S, Russell CG, Campbell K, Laws R, Ong KL (2017). Assessing user engagement of an mHealth intervention: development and implementation of the Growing Healthy app engagement index. JMIR Mhealth Uhealth.

[41] Amoakoh HB, Klipstein-Grobusch K, Ansah EK, Grobbee DE, Yveoo L, Agyepong I (2019). How and why front-line health workers (did not) use a multifaceted mHealth intervention to support maternal and neonatal healthcare decision-making in Ghana. BMJ Glob Health.

[42] Maaløe N, Ørtved AM, Sørensen JB, Sequeira Dmello B, van den Akker T, Kujabi ML (2021). The injustice of unfit clinical practice guidelines in low-resource realities. Lancet Glob Health.

[43] Main EK, Cape V, Abreo A, Vasher J, Woods A, Carpenter A (2017). Reduction of severe maternal morbidity from hemorrhage using a state perinatal quality collaborative. Am J Obstet Gynecol.

[44] Ye J, He L, Beestrum M (2023). Implications for implementation and adoption of telehealth in developing countries: a systematic review of China's practices and experiences. NPJ Digit Med.

