# Supplementary material for: Telehealth usefulness during postpartum hemorrhage management: a systematic review
Source: Rev Bras Ginecol Obstet. 2026 Sep 23;48:e-rbgo71. doi: 10.61622/rbgo/2026rbgo71 (PMC13690380; doi:10.61622/rbgo/2026rbgo71)
Supplement: Supplementary file 1 [file 1806-9339-rbgo-48-e-rbgo71-Suppl01.pdf]

Supplementary material

Chart 1S. MEDLINE (Ovid) Search Strategy

Source:	Ovid MEDLINE(R) ALL <1946 to March 26, 2024>	
**#**	**Search**	**Results**
1	exp Telemedicine/	47030
2	(tele adj3 (medicine* or health* or care* or monitor*)).ab,kf,ti.	1377
3	(Mhealth or eHealth or "personal digital assistant" or (mobile adj1 (health* or apps health or phone* or device*))).ab,kf,ti.	38351
4	or/1-3 [Telemedicine]	77760
5	Postpartum Period/	31985
6	Postpartum Hemorrhage/	8693
7	(((postpartum or puerperium) adj3 (h?emorrh* or bleed* or period*)) or PPH).ab,kf,ti.	26739
8	or/5-7 [Postpartum Hemorrhage]	55600
9	Pregnancy/	1008712
10	exp Pregnancy Complications/	482391
11	exp Obstetric Labor Complications/	82224
12	((pregnan* or labo?r) adj3 complication*).ab,kf,ti.	35569
13	or/9-12 [Pregnancy complications]	1049467
14	8 or 13 [Postpartum Hemorrhage / Pregnancy complications]	1064538
15	and/4,14 [Telemedicine & Postpartum Hemorrhage / Pregnancy complications]	2129

Chart 2S. Embase (Ovid) Search Strategy

Source:	Embase Classic+Embase <1947 to 2024 March 26>	
**#**	**Search**	**Results**
1	exp telemedicine/	76069
2	(tele adj3 (medicine* or health* or care* or monitor*)).ab,kw,ti.	2197
3	(Mhealth or eHealth or "personal digital assistant" or (mobile adj1 (health* or apps health or phone* or device*))).ab,kw,ti.	39075
4	or/1-3 [Telemedicine]	109843
5	puerperium/	57885
6	exp postpartum hemorrhage/	20681
7	(((postpartum or puerperium) adj3 (h?emorrh* or bleed* or period*)) or PPH).ab,kw,ti.	35062
8	or/5-7 [Postpartum Hemorrhage]	87843
9	pregnancy/	845047
10	exp pregnancy complication/	626141
11	exp labor complication/	257956
12	((pregnan* or labo?r) adj3 complication*).ab,kw,ti.	33614
13	or/9-12 [Pregnancy complications]	1230695
14	8 or 13 [Postpartum Hemorrhage / Pregnancy complications]	1256798
15	and/4,14 [Telemedicine & Postpartum Hemorrhage / Pregnancy complications]	2912

Chart 3S. Cochrane Library Search Strategy

Source:	Cochrane	
**#**	**Search**	**Results**
#1	MeSH descriptor: [Telemedicine] explode all trees	4786
#2	(tele NEAR/3 (medicine* or health* or care* or monitor*)):ab,kw,ti	518
#3	(Mhealth or eHealth or "personal digital assistant" or (mobile NEAR/1 (health* or apps health or phone* or device*))):ab,kw,ti	10299
#4	#1 OR #2 OR #3	14158
#5	MeSH descriptor: [Postpartum Period] explode all trees	2772
#6	MeSH descriptor: [Postpartum Hemorrhage] explode all trees	986
#7	(((postpartum or puerperium) NEAR/3 (h?emorrh* or bleed* or period*)) or PPH):ab,kw,ti	6499
#8	#5 OR #6 OR #7	7228
#9	MeSH descriptor: [Pregnancy] this term only	33215
#10	MeSH descriptor: [Pregnancy Complications] explode all trees	17652
#11	MeSH descriptor: [Obstetric Labor Complications] explode all trees	5785
#12	((pregnan* or labo?r) NEAR/3 complication*):ab,kw,ti	7892
#13	#9 OR #10 OR #11 OR #12	39481
#14	#8 OR #13	42934
#15	#4 AND #14	559
#16	#15 in Cochrane Reviews	6
#17	#15 in Trials	553

	**Results**
Cochrane Database of Systematic Reviews	6
Issue 3 of 12, March 2024	
Cochrane Central Register of Controlled Trials	553
Issue 2 of 12, February 2024	
	559
